# Gene set enrichment analysis of pathophysiological pathways highlights oxidative stress in psychosis

**DOI:** 10.1038/s41380-022-01779-1

**Published:** 2022-09-21

**Authors:** Giorgio Pistis, Javier Vázquez-Bourgon, Margot Fournier, Raoul Jenni, Martine Cleusix, Sergi Papiol, Sophie E. Smart, Antonio F. Pardiñas, James T. R. Walters, James H. MacCabe, Zoltán Kutalik, Philippe Conus, Benedicto Crespo-Facorro, Kim Q Do

**Affiliations:** 1 Department of Psychiatry, Lausanne University Hospital and University of Lausanne, Prilly, Lausanne, Switzerland; 2 Centro de Investigación Biomédica en Red de Salud Mental (CIBERSAM), Instituto de Salud Carlos III, Madrid, Spain; 3 Department of Psychiatry, University Hospital Marqués de Valdecilla, Instituto de Investigación Marqués de Valdecilla (IDIVAL), Santander, Spain; 4 Departamento de Medicina y Psiquiatría, Universidad de Cantabria, Santander, Spain; 5 Unit for Research in Schizophrenia, Center for Psychiatric Neuroscience, Department of Psychiatry, Lausanne University Hospital (CHUV), Prilly, Lausanne, Switzerland; 6 Institute of Psychiatric Phenomics and Genomics (IPPG), University Hospital, LMU Munich, Munich, Germany; 7 Department of Psychiatry and Psychotherapy, University Hospital, LMU Munich, Munich, Germany; 8 MRC Centre for Neuropsychiatric Genetics and Genomics, Division of Psychological Medicine and Clinical Neurosciences, Cardiff University, Cardiff, UK; 9 Department of Psychosis Studies, Institute of Psychiatry, Psychology and Neuroscience, King’s College London, London, UK; 10 University Center for Primary Care and Public Health, University of Lausanne, Lausanne, Switzerland; 11 Department of Computational Biology, University of Lausanne, Lausanne, Switzerland; 12 Swiss Institute of Bioinformatics, Lausanne, Switzerland; 13 Service of General Psychiatry, Department of Psychiatry, University Hospital Center and University of Lausanne, Prilly, Lausanne, Switzerland; 14 Virgen del Rocío University Hospital, Institute of Biomedicine of Seville (IBiS)-CSIC, University of Seville, First-episode Psychosis Research Network of Andalusia (Red PEPSur), Seville, Spain

**Keywords:** Genetics, Schizophrenia

## Abstract

Polygenic risk prediction remains an important aim of genetic association studies. Currently, the predictive power of schizophrenia polygenic risk scores (PRSs) is not large enough to allow highly accurate discrimination between cases and controls and thus is not adequate for clinical integration. Since PRSs are rarely used to reveal biological functions or to validate candidate pathways, to fill this gap, we investigated whether their predictive ability could be improved by building genome-wide (GW-PRSs) and pathway-specific PRSs, using distance- or expression quantitative trait loci (eQTLs)- based mapping between genetic variants and genes. We focused on five pathways (glutamate, oxidative stress, GABA/interneurons, neuroimmune/neuroinflammation and myelin) which belong to a critical hub of schizophrenia pathophysiology, centred on redox dysregulation/oxidative stress. Analyses were first performed in the Lausanne Treatment and Early Intervention in Psychosis Program (TIPP) study (*n* = 340, cases/controls: 208/132), a sample of first-episode of psychosis patients and matched controls, and then validated in an independent study, the epidemiological and longitudinal intervention program of First-Episode Psychosis in Cantabria (PAFIP) (*n* = 352, 224/128). Our results highlighted two main findings. First, GW-PRSs for schizophrenia were significantly associated with early psychosis status. Second, oxidative stress was the only significantly associated pathway that showed an enrichment in both the TIPP (*p* = 0.03) and PAFIP samples (*p* = 0.002), and exclusively when gene-variant linking was done using eQTLs. The results suggest that the predictive accuracy of polygenic risk scores could be improved with the inclusion of information from functional annotations, and through a focus on specific pathways, emphasizing the need to build and study functionally informed risk scores.

## Introduction

Schizophrenia is a chronic and, in some cases, disabling mental disorder characterized by disturbances in thought, perception, emotion and behavior [1]. Schizophrenia affects around 0.7% of the population [2] and genetic studies have provided evidence of its high heritability (41–87%) [3] and polygenicity [4]. In recent years, the emergence of well-powered genome-wide association studies (GWASs) has provided novel insights into the etiology of schizophrenia and shown that many common genetic variants contribute to the risk of developing schizophrenia [4–7]. Based on these GWAS results, a growing literature has examined polygenic risk scores (PRSs) as indices of genetic risk for schizophrenia and found that PRSs were able to differentiate individuals diagnosed with schizophrenia from unaffected individuals at a group-level but only explain 5.7% of variance in case-control status (on the liability scale) [4]. Recently, PRS have been used to predict the risk of being diagnosed with schizophrenia after having a first-episode of psychosis, demonstrating one use, theoretically, for PRSs in clinical health care settings [8, 9]. Despite these consistent and statistically robust findings, the effects of the PRSs were not large enough to allow high-accuracy discrimination of cases and controls and consequently, not yet adequate to assist with clinical decision making on a case-by-case basis [10]. Nevertheless, risk prediction remains one of the primary aims of genetic studies [11] and the question remains whether PRSs could be used, in the future, for early intervention and targeted preventions. The improvement of the predictive accuracy, and therefore clinical utility of PRSs, may depend on several factors, but two important developments include: focusing on alleles within specific biological pathways or gene sets associated with the disease of interest and the prioritization of functional variants.

**Fig. 1:**
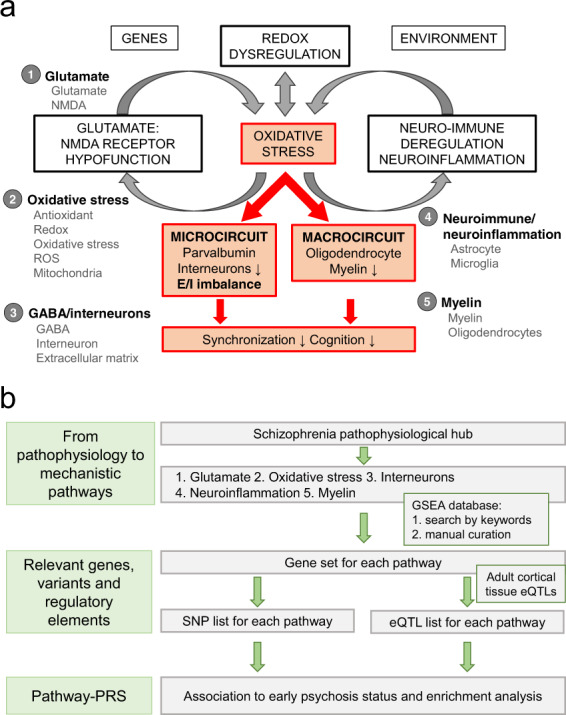
Workflow analysis. Panel **a** shows a schematic representation of the concept proposed in this paper, showing the reciprocal interaction between mitochondria, NMDAR, neuro-immune system, dopamine on one hand and the complex redox regulation/oxidative stress on the other. We focused on five pathways (glutamate, oxidative stress, GABA/interneurons (hereafter called interneurons), neuroimmune/neuroinflammation (hereafter called neuroinflammation) and myelin). Panel **b** summarizes the experimental set up used for five different pathways. For each pathway, we defined the corresponding biological mechanisms and used them as keywords that were entered into the GSEA platform to retrieve the corresponding gene sets. The gene sets were then manually parsed to keep only those more pertinent to each pathway. For each gene set we defined (1) those SNPs that mapped to the gene set and (2) those SNPs that were eQTLs at least for one gene of the set.

In terms of biological pathways associated with schizophrenia, converging evidence from clinical and preclinical data highlights the interaction between genetic and environmental risks that leads to dysfunction during development in NMDAR-mediated signalling, neuroimmune regulation/neuroinflammation, and mitochondrial function. This dysfunction initiates “vicious circles” centred on redox dysregulation/oxidative stress as one critical hub of schizophrenia pathophysiology [12]. In addition, impairments of the maturation and function of local parvalbumin-GABAergic interneuron microcircuits and myelinated fibres of long-range macrocircuitry are thought to cause the neural circuit synchronization abnormalities and cognitive, emotional, social and sensory deficits characteristic of schizophrenia. Therefore, in this study we considered the following pathophysiological pathways: (1) glutamate [13–15], (2) oxidative stress [12, 16], (3) GABA/interneurons (hereafter called interneurons) [17, 18], (4) neuroimmune/neuroinflammation (hereafter called neuroinflammation) [19–22] and (5) myelin [23] (Fig. 1a).

In terms of functional variants, GWAS hits are found to be enriched in regulatory sequences. These variants do not directly affect the coding sequence of a gene, suggesting that they may play a fundamental role in disease by regulating the expression levels or by affecting the splicing of genes instead [24–28]. Variants that influence gene expression are known as expression quantitative trait loci (eQTLs).

Usually, PRSs do not account for biological functions nor focus on candidate pathways, therefore, our aim was to investigate whether the predictive ability of the schizophrenia PRS can be improved by building genome-wide and pathway-specific PRSs using single nucleotide polymorphisms (SNPs) and eQTLs (Fig. 1b) in two first-episode psychosis case-control samples.

## Materials and methods

Analyses were first conducted in a sample of patients recruited during a first-episode of psychosis and ancestry-matched control subjects from the city of Lausanne (TIPP study), and then validated in an independent first-episode of psychosis cohort from the autonomous region of Cantabria in northern Spain (PAFIP study) (Supplementary Table 1).

### Participants

#### TIPP

Participants were recruited from the Treatment and Early Intervention in Psychosis Program (TIPP), which offers 3 years of treatment to patients aged 18–35 years [29]. Entry criteria to the program are: (1) aged between 18 and 35, (2) residing in the catchment area (Lausanne and surroundings; population about 300,000), (3) meeting threshold criteria for psychosis, as defined by the “Psychosis threshold” subscale of the Comprehensive Assessment of At Risk Mental States (CAARMS [30]) Scale [29], (4) no psychosis related to intoxication or organic brain disease and (5) intelligence quotient ≥70. Diagnosis was based on the Diagnostic and Statistical Manual of Mental Disorders, Fourth Edition (DSM-IV) [31] and determined by expert consensus between a senior psychiatrist and a senior psychologist, who reviewed patient files and also determined the date a participant first met the threshold criteria for psychosis. Duration of illness was defined as the time between reaching the psychosis threshold for the first time and the time of assessment. Healthy controls, recruited from similar geographic and sociodemographic areas through advertisement, were assessed by the Diagnostic Interview for Genetic Studies [32] and matched on gender, age and handedness. Major mood, psychotic or substance-use disorder as well as having a first-degree relative with a psychotic disorder were exclusion criteria for controls. The sample for the present study comprised 339 patients and 168 controls. All the participants in this study gave written informed consent in accordance with our institutional guidelines (study and consent protocol approved by the Local Ethical Committee: *“Commission cantonale d’éthique de la recherché sur l’être humain – CER-VD*). The present analysis involves participants included in the TIPP program between 2007 and the end of 2019 [11].

#### PAFIP

PAFIP is an epidemiological and longitudinal intervention program of First-Episode Psychosis in Cantabria [33]. All referrals to PAFIP between March 2001 and December 2014 were screened following these inclusion criteria: (1) aged between 16 and 60 years, (2) living in the catchment area, (3) experiencing their first-episode of psychosis and meeting DSM-IV criteria for a diagnosis of schizophreniform disorder, schizophrenia, schizoaffective disorder, brief reactive psychosis, or psychosis not otherwise specified and (4) no prior treatment with antipsychotic medication or, if previously treated, a total life-time of adequate antipsychotic treatment of less than 6 weeks. DSM-IV criteria for drug or alcohol dependence, intellectual disability and having a history of neurological disease or head injury were regarded as exclusion criteria. The diagnoses were confirmed through the administration of the Structured Clinical Interview for DSM-IV (SCID–I) [34], conducted by an experienced psychiatrist six months after the baseline visit. A personal or family history of mental disorder were exclusion criteria for healthy controls, who were recruited from the same geographical area. The sample for the present study comprised 268 patients, for whom combined genetic and psychiatric data were available, and 139 controls. All subjects provided written informed consent prior to their inclusion in the study, which was approved by the regional ethics committee (Clinical Research Ethics Committee of Cantabria).

### Genetic data

#### TIPP

Genome-wide genotyping was performed in two batches using the Infinium OmniExpress-24 v1.3 SNP array. Nuclear DNA was extracted from whole blood of all participants. Genotypes from both batches were called using GenomeStudio Software [35]. Both batches underwent the same quality controls and imputation procedures. Batch 1 included 266 patients and batch 2 included 241 individuals (73 patients + 168 controls). Duplicate individuals, and first and second degree relatives, were identified and then removed by computing pair-wise genomic kinship coefficients, using KING [36]. Subjects were excluded from the analysis in case of a genotype call rate less than 95%. To account for possible population stratification, we computed principal component analysis (PCA) using PLINK [37] with default options and excluded individuals who did not segregate with European samples based on principal component analysis. A total of 165 patients on batch 1 and 175 individuals (43 patients + 132 controls) on batch 2 passed QC thresholds. Quality control for single nucleotide polymorphisms (SNPs) was performed using the following criteria: monomorphic (or with minor allele frequency (MAF) < 1%), call rates less than 95%, deviation from the Hardy-Weinberg equilibrium (HWE) (*p* < 1 × 10^−6^). Phased haplotypes were generated using SHAPEIT2 [38, 39]. Imputation was performed using minimac3 [40] and the Haplotype Reference Consortium (HRC version r1.1) [41] hosted on the Michigan Imputation Server [40]. We used imputed allele dosages for all SNPs to avoid genotyping missingness. A MAF > 1% and an imputation quality Rsq >0.3 was required for the inclusion of the variants into further analyses. In order to identify, and eventually reduce, any batch effect introduced by the two genotyping batches, we performed a negative control GWAS where the outcome was defined as the batch membership (“control” = batch1, “case” = batch2) and using cases only to avoid removing true association signals. In this way we could identify and remove 566 SNPs (at a false discovery rate (FDR) < 5%), which showed significant difference in allele frequency between the batches.

#### PAFIP

Genome-wide genotyping was performed using the Illumina Infinium PsychArray. Nuclear DNA was extracted from whole blood of all participants. Genotypes were called using GenomeStudio Software [35]. The original sample consisted of 407 samples (268 patients + 139 controls). SNPs and individuals were excluded if their call rate was below 98%. Likewise, SNPs with MAF < 0.5% were removed. Participants whose genetic sex did not match self-reported sex in the clinical documentation were excluded. Duplicate samples and first- and second-degree relatives, were identified and then removed after computing their pairwise identity-by-descent values with PLINK [23]. To account for possible population stratification, we computed MDS components using PLINK [23] with default options and excluded individuals who did not segregate with European samples based on principal component analysis. Subjects with heterozygosity value >3.81 SD were also removed. SNPs with a HWE *p* value <1 × 10^−4^ or a MAF < 1% were excluded, followed by palindromic SNPs and SNPs with a MAF deviation >10% with respect to EUR reference populations. A total of 359 samples passed quality control. Prephasing and imputation were performed using, respectively, eagle [42] and Minimac4 [26] and the Haplotype Reference Consortium (HRC version r1.1) [27] hosted on the Michigan Imputation Server [26]. We used imputed allele dosages for all SNPs to avoid genotyping missingness. A MAF > 1% and an imputation quality Rsq >0.3 was required for the inclusion of the variants into further analyses.

### Genome-wide and pathway-specific polygenic risk scores

An overview of the experimental setup describing all the steps from the pathophysiological hub to the calculation of the risk scores is shown in Fig. 1b. In total, we derived eighteen polygenic risk scores (PRSs): three genome-wide risk scores (GW-PRSs) and fifteen pathway-specific risk scores (pathway-PRSs). PRS differed in terms of which variants were included: (1) single nucleotide polymorphisms (SNPs), (2) expression quantitative trait loci (eQTLs) from the GTEx database or (3) and eQTLs from the MetaBrain database (see methods paragraph “Expression quantitative trait loci (eQTLs) databases”). For the GW-PRSs, we either used all the SNPs available in our dataset (GW-PRS_SNPs_), all the eQTLs listed in the GTEx database (GW-PRS_eQTLs_), or all the eQTLs listed in the MetaBrain database (GW-PRS_eQTLs_). For the pathway-PRSs, we identified five pathways and the genes included within those pathways (see methods paragraph “Pathways selection”). For the pathway-PRSs, we either used the SNPs which were mapped to each of the pathways (inclusive of a 50-kb flanking buffer), the eQTLs, listed in the GTEx database, associated with genes that mapped to the pathways, or the eQTLs, listed in the MetaBrain database, associated with genes that mapped to the pathways.

### Pathways selection

We focused on five pathophysiological pathways of interest [12]: (1) glutamate, (2) oxidative stress, (3) interneurons, (4) neuroinflammation and (5) myelin. For each pathway, we defined the corresponding biological mechanisms and used them as keywords that were entered into the GSEA platform to retrieve the corresponding gene sets [43]. The keywords we defined are: (1) glutamate, NMDA for the glutamate pathway, (2) antioxidant, redox, oxidative stress, ROS, mitochondria for the oxidative stress pathway, (3) GABA, interneuron, extracellular matrix for interneurons pathway, (4) astrocyte, microglia for the neuroinflammation pathway and (5) myelin, oligodendrocytes for the myelin pathway (Fig. 1a). The gene sets were then manually parsed to keep only those pertinent to each pathway. We then merged the gene sets belonging to the same pathway, and found 627, 1355, 1347, 1657 and 195 genes for pathways 1 to 5 respectively (Supplementary Tables 2,5-9).

### Expression quantitative trait loci (eQTLs) databases

Functional variants used to derive GW-PRS_eQTLs_ and pathway-PRS_eQTLs_ were identified through two different databases: (1) Genotype-Tissue Expression v8 (GTEx) [44] and (2) MetaBrain [45]. In each database, we considered only cis-eQTLs of the adult brain cortex tissue (cis-eQTLs were defined as SNPs that reside within 1 Mb of the transcription start site) and only used European samples (GTEx v8: *n* = 250, MetaBrain: *n* = 2970). Before calculating the risk scores, we filtered the GTEx and MetaBrain databases in order to keep only those eQTLs that showed a nominally significant association (*p* value <0.05) with any gene at the genomic level (GW-PRS_eQTLs_) or at least with one of the selected genes at pathway level (pathway-PRS_eQTLs_).

### Polygenic risk scores calculation

Polygenic risk scores were derived using the “standard weighted allele” method implemented in PRSice-2 [46], using standardized effect sizes from a large GWAS on schizophrenia that included mostly individuals of European descent [4]. Linkage disequilibrium (LD) clumping was performed to retain only data for independent SNPs (r2 < 0.1, 1 Mb window). For GW-PRSs, in the main analyses, we applied a GWAS *p* value threshold (pt) ≤ 0.05, as previous work suggests that this is the optimum threshold for discriminating between schizophrenia cases and controls [5]. We also performed a sensitivity analysis using a pt ≤ 1. For each pathway-PRS, we used PRSet [46] to calculate a competitive *p* value which indicates its level of enrichment over a random set of SNPs of the same size. We performed 10,000 permutations for each pathway-PRS and counted how many random set of SNPs (x) outperformed the association strength of the pathway-PRS with early psychosis case-control status. We then calculated competitive *p* values as x/10,000 to be able to obtain *p* values as low as 1 × 10^−4^. By default, PRSet derives pathway-PRSs at pt ≤ 1, to avoid the PRS containing only a small portion of SNPs within the pathway, which can happen when more stringent pt thresholds are used. In the main pathway-PRSs analysis, we applied a pt ≤ 1 as suggested by PRSet authors, and, in a sensitivity analysis, applied a pt ≤ 0.05.

### Statistical analysis

Case-control status (dependent variable) was regressed on the polygenic risk scores (GW-PRSs and pathway-PRSs) using logistic regressions and the first five ancestry-informative genetic principal components were included as covariates. The variance explained by the PRS (Nagelkerke r^2^) was calculated by subtracting the r^2^ of the null model (containing only the covariates) to the r^2^ of the full model (containing PRS + covariates). The variance explained by the PRS was transformed to a liability scale, using the r^2^ coefficient proposed by Lee et al. [47] and a population prevalence of 0.7%. The area under the receiver operator characteristic curve (AUROC) was calculated in a model with no covariates using the pROC R package [48]. For the analyses involving the eighteen PRSs, the significance level was set to *p* = 0.0027 (0.05/18) according to the Bonferroni correction for multiple testing.

## Results

A total of 692 participants from 2 separate studies were included in the analysis; 259 were women (37.4%) and the mean (SD) age at study interview was 29.5 (9.15) years.

### Genome-wide polygenic risk scores prediction

**Fig. 2:**
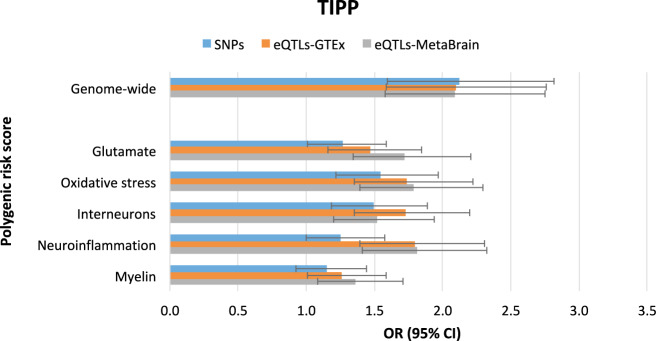
Results for the TIPP study and polygenic risk scores (GW-PRSs and pathway-PRSs) analyses at pt ≤ 1. Early psychosis status (dependent variable) was regressed on the polygenic risk scores using logistic regressions and including the first five ancestry-informative genetic principal components as covariates. Horizontal bars show the Odds Ratio estimates (OR), and error bars indicate 95% confidence intervals (95% CI).

**Fig. 3:**
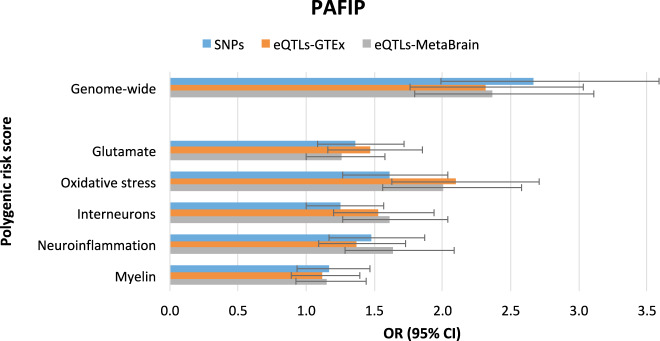
Results for the PAFIP study and polygenic risk scores (GW-PRSs and pathway-PRSs) analyses at pt ≤ 1. Early psychosis status (dependent variable) was regressed on the polygenic risk scores using logistic regressions and including the first five ancestry-informative genetic principal components as covariates. Horizontal bars show the Odds Ratio estimates (OR), and error bars indicate 95% confidence intervals (95% CI).

In the TIPP sample, GW-PRSs were significantly associated with early psychosis case-control status with similar odds ratios for GW-PRS_SNPs_, GW-PRS_eQTLs_ based on GTEx, and GW-PRS_eQTLs_ based on MetaBrain (OR = 2.12, 95% CI = 1.61–2.81, OR = 2.10, 95% CI = 1.60–2.75 and OR = 2.06, 95% CI = 1.56–2.70, respectively; Supplementary Table 3 and Supplementary Figure 1). Similarly, in the PAFIP sample, GW-PRSs were significantly associated with early psychosis case-control status using GW-PRS_SNPs_, GW-PRS_eQTLs_ based on GTEx, and GW-PRS_eQTLs_ based on MetaBrain (OR = 2.73, 95% CI = 2.03–3.67, OR = 2.30, 95% CI = 1.76–3.02 and OR = 2.27, 95% CI = 1.72–2.98, respectively; Supplementary Table 4 and Supplementary Figure 2). The GW-PRSs predictive power and the variance explained by the polygenic scores on the liability scale were also similar within each sample (Supplementary Tables 3-4 and Supplementary Figs. 1–2). Sensitivity analyses using GW-PRSs with a pt ≤ 1 showed a similar pattern and, as expected, were significantly associated with early psychosis status (Tables 1–2 and Figs. 2–3).

**Table 1:** Results for the TIPP study and polygenic risk scores (GW-PRSs and pathway-PRSs) analyses at pt ≤ 1.

		OR	CI (95%)	Pval	Competitive Pval	N SNPs	Nagelkerke r^2^	Liability-scale r^2^	AUROC
eQTLs-GTEx	Genome-wide	2.10	[1.59–2.76]	1.51E-07 *	–	169,272	0.117	0.043	0.67
	Glutamate	1.47	[1.16–1.85]	1.25E-03 *	0.129	15,215	0.042	0.018	0.59
	Oxidative stress	1.73	[1.35–2.22]	1.30E-05 *	0.033 **	30,465	0.079	0.030	0.64
	Interneurons	1.73	[1.35–2.20]	1.01E-05 *	0.020 **	26,644	0.080	0.029	0.65
	Neuroinflammation	1.79	[1.39–2.31]	6.98E-06 *	0.032 **	35,338	0.084	0.034	0.65
	Myelin	1.26	[1.01–1.59]	0.044	0.263	5,404	0.016	0.008	0.56
eQTLs-MetaBrain	Genome-wide	2.08	[1.58–2.75]	2.17E-07 *	–	170,793	0.115	0.042	0.66
	Glutamate	1.72	[1.34–2.21]	1.87E-05 *	0.014 **	19,946	0.077	0.032	0.64
	Oxidative stress	1.79	[1.40–2.29]	4.31E-06 *	0.036 **	40,014	0.087	0.029	0.66
	Interneurons	1.52	[1.20–1.94]	6.14E-04 *	0.428	39,163	0.048	0.017	0.62
	Neuroinflammation	1.81	[1.41–2.33]	3.58E-06 *	0.042 **	45,940	0.089	0.031	0.66
	Myelin	1.36	[1.08–1.71]	8.92E-03	0.129	6,867	0.027	0.013	0.59
SNPs	Genome-wide	2.12	[1.60–2.82]	2.03E-07 *	–	241,338	0.115	0.041	0.66
	Glutamate	1.27	[1.01–1.59]	0.042	0.658	17,115	0.016	0.005	0.58
	Oxidative stress	1.55	[1.22–1.97]	3.72E-04 *	0.169	25,186	0.052	0.018	0.62
	Interneurons	1.50	[1.18–1.89]	7.09E-04 *	0.274	28,931	0.046	0.017	0.62
	Neuroinflammation	1.25	[1.00–1.57]	0.052	0.903	30,046	0.015	0.003	0.56
	Myelin	1.15	[0.92–1.44]	0.219	0.574	5,375	0.006	0.004	0.54

Early psychosis status (dependent variable) was regressed on the PRSs using logistic regressions and including the first five ancestry-informative genetic principal components as covariates. Odds ratios (OR), 95% confidence intervals CI (95%) and *p* value (Pval) show the predictive power of each PRS. Associations surviving correction for multiple testing alpha level of 0.0027 (0.05/18) are denoted with an asterisk (*). Number of SNPs included in the pathway analysis shows how many SNPs are included in each PRS. The column Competitive P val shows the enrichment after 10,000 permutations. Significant enrichments are denoted with two asterisks (**). Nagelkerke r2 shows the variance explained by the PRS. Liability-scale r2 shows the variance explained by the PRS using a population prevalence of 0.7%. AUROC shows the area under the receiver operating characteristic curve.

**Table 2:** Results for the PAFIP study and polygenic risk scores (GW-PRSs and pathway-PRSs) analyses at pt ≤ 1.

		OR	CI (95%)	Pval	Competitive Pval	N SNPs	Nagelkerke r^2^	Liability-scale r^2^	AUROC
eQTLs-GTEx	Genome-wide	2.31	[1.76–3.03]	1.37E-09 *	–	167,745	0.156	0.106	0.70
	Glutamate	1.47	[1.16–1.85]	1.25E-03 *	0.256	14,733	0.040	0.021	0.60
	Oxidative stress	2.10	[1.63–2.71]	1.29E-08 *	1.70E-03 **	29,803	0.132	0.048	0.68
	Interneurons	1.53	[1.20–1.94]	5.68E-04 *	0.456	25,923	0.047	0.029	0.61
	Neuroinflammation	1.37	[1.09–1.73]	7.58E-03	0.914	34,540	0.027	0.018	0.57
	Myelin	1.11	[0.89–1.39]	0.350	0.766	5,242	0.003	0.002	0.48
eQTLs-MetaBrain	Genome-wide	2.36	[1.79–3.10]	8.58E-10 *	–	169,359	0.161	0.103	0.70
	Glutamate	1.26	[1.00–1.58]	0.049	0.869	19,429	0.015	0.007	0.56
	Oxidative stress	2.00	[1.56–2.58]	6.71E-08 *	0.022 **	39,323	0.119	0.054	0.67
	Interneurons	1.61	[1.27–2.04]	1.01E-04 *	0.509	38,260	0.059	0.042	0.63
	Neuroinflammation	1.63	[1.28–2.09]	8.01E-05 *	0.618	44,953	0.062	0.028	0.64
	Myelin	1.15	[0.92–1.44]	0.205	0.719	6,674	0.006	0.003	0.54
SNPs	Genome-wide	2.67	[1.99–3.59]	7.11E-11 *	–	239,925	0.191	0.140	0.72
	Glutamate	1.36	[1.08–1.72]	9.16E-03	0.576	16,713	0.026	0.013	0.57
	Oxidative stress	1.61	[1.27–2.04]	9.87E-05 *	0.225	24,630	0.059	0.024	0.63
	Interneurons	1.25	[1.00–1.57]	0.052	0.967	28,290	0.014	0.012	0.56
	Neuroinflammation	1.47	[1.16–1.87]	1.32E-03 *	0.643	28,986	0.040	0.021	0.60
	Myelin	1.17	[0.93–1.47]	0.175	0.600	5,145	0.007	0.021	0.54

Early psychosis status (dependent variable) was regressed on the PRSs using logistic regressions and including the first five ancestry-informative genetic principal components as covariates. Odds ratios (OR), 95% confidence intervals CI (95%) and *p* value (Pval) show the predictive power of each PRS. Associations surviving correction for multiple testing alpha level of 0.0027 (0.05/18) are denoted with an asterisk (*). Number of SNPs included in the pathway analysis shows how many SNPs are included in each PRS. The column Competitive Pval shows the enrichment after 10,000 permutations. Significant enrichments are denoted with two asterisks (**). Nagelkerke r2 shows the variance explained by the PRS. Liability-scale r2 shows the variance explained by the PRS using a population prevalence of 0.7%. AUROC shows the area under the receiver operating characteristic curve.

### Pathway-specific polygenic risk scores prediction

Pathway-PRS_SNPs_ did not show any significant enrichment in either the TIPP or the PAFIP samples. In the TIPP sample, pathway-PRS_eQTLs_ based on GTEx showed an enrichment for the oxidative stress, interneurons and neuroinflammation pathways (associated to early psychosis case-control status respectively: OR = 1.73, 95% CI = 1.35–2.22, OR = 1.73, 95% CI = 1.35–2.20 and OR = 1.79, 95% CI = 1.39–2.31), whereas analyses based on MetaBrain showed an enrichment for the glutamate, oxidative stress and neuroinflammation pathways (associated to early psychosis case-control status respectively: OR = 1.72, 95% CI = 1.34–2.21, OR = 1.79, 95% CI = 1.40–2.29 and OR = 1.81, 95% CI = 1.41–2.33) (Table 1 and Fig. 2). In the PAFIP sample, pathway-PRS_eQTLs_ based on both GTEx and MetaBrain showed an enrichment for the oxidative stress pathway (associated to early psychosis case-control status respectively: OR = 2.10, 95% CI = 1.63–2.71 and OR = 2.00, 95% CI = 1.56–2.58) (Table 2 and Fig. 3). In the TIPP study, the polygenic variance of oxidative stress pathway-PRS_eQTLs_ on the liability scale in case-control status, was 3.0% and 2.9% for GTEx and MetaBrain respectively, accounting in each database for 69.8% of the polygenic variance on the liability scale of the respective GW-PRS_eQTLs_. In PAFIP study, the polygenic variance of oxidative stress pathway-PRS_eQTLs_ on the liability scale in case-control status, was 4.8% and 5.4% for GTEx and MetaBrain databases, accounting for 45.2% and 52.0% of the polygenic variance on the liability scale of the respective GW-PRS_eQTLs_. In the TIPP study, the AUROC of the oxidative stress pathway-PRS_eQTLs_ on the case-control status was 0.64 and 0.66 for GTEx and MetaBrain respectively, accounting for 96% and 100% of the predictive power of the two GW-PRS_eQTLs_ calculated on the same databases. In PAFIP study, the AUROC of the oxidative stress pathway-PRS_eQTLs_ was 0.68 and 0.67 for GTEx and MetaBrain databases, accounting for 97% and 95% of the predictive power of the two GW-PRS_eQTLs_ calculated on the same databases. Sensitivity analyses showed a similar pattern (Supplementary Tables 3-4 and Supplementary Figs. 1–2).

## Discussion and conclusion

The present study is, to our knowledge, the first to investigate the ability of both genome-wide (GW-PRSs) and pathways (pathway-PRSs) schizophrenia polygenic risk scores to discriminate early psychosis case-control status. In addition, we compared PRS derived using SNPs and brain cortex eQTLs. We found that GW-PRSs were significantly associated with the early psychosis status regardless of whether SNPs or eQTLs were used. In addition, the only pathway based PRS that showed a replicated association with early psychosis status was the oxidative stress pathway derived using eQTLs.

Although all the GW-PRSs could predict the early psychosis status, the GW-PRS_SNPs_ showed slightly stronger association, probably due to a higher number of genetic variants (~ 31.9% more genetic variants compared to GW-PRS_eQTLs_).

We focused on five pathways (glutamate, oxidative stress, interneurons, neuroinflammation and myelin) which belong to a “central hub” in schizophrenia pathophysiology [12]. Among the pathway-PRSs tested, we only found enrichment for the oxidative stress pathway-PRS, and only when exclusively using functional SNPs. This supports the idea that redox dysregulation/oxidative stress plays a critical role in pathophysiology of schizophrenia [12, 18, 49, 50].

Notably, in the TIPP study, the predictive power of oxidative stress pathway-PRS_eQTLs_ on the case-control status, accounted for up to 100% of the predictive power of the respective GW-PRS_eQTLs_, whereas in the PAFIP study, the predictive power, accounted up to 97% of the predictive power of the respective GW-PRS_eQTLs_.

This highlights the critical role of cis-regulatory elements eQTLs, both genome-wide and within the oxidative stress pathway, which are potentially driven by gene-environment interactions. Elam et al. in 2019 first reported how risk scores, computed from functional candidate SNPs mapped to genes, may be more predictive than data-driven approach PRSs when examining childhood aggression as the trait of interest [51]. Here, instead of only analyzing pre-determined pathways or gene sets derived from databases, we took advantage of the existing literature which has identified a “central hub” where genetic and environmental risks converge, and are thought to be involved in schizophrenia.

Experimental and translational evidences highlight the crucial role of either Glutamate/NMDAR hypofunction [13, 14] or neuroimmune dysregulation [20, 21]/ neuroinflammation [22], initiating “vicious circles” centred on oxidative stress during neurodevelopment [12]. These processes would amplify one another in positive feed-forward loops, leading to persistent impairments of the maturation and function of local parvalbumin-GABAergic neurons microcircuits and myelinated fibres of long-range macrocircuitry. This is at the basis of neural circuit synchronization impairments and cognitive, emotional, social and sensory deficits characteristic of schizophrenia [12, 16, 18, 52]. Our findings support the proposal that the interaction of genes and environment within these functional pathways is a pathophysiological mechanism which leads to the emergence of schizophrenia, placing the emphasis on oxidative stress.

The results of the present study need to be viewed in the light of several limitations. Firstly, the limited sample sizes in the two studies could have led to reduced statistical power, low accuracy of discriminative ability (AUROC) and an inability to detect true associations of small effect sizes (i.e. through simulations we found that the statistical power of PAFIP to replicate the association found in TIPP on oxidative stress pathway-PRS_eQTLs_ is 49% and it would require a sample size of 500 to reach 81%). Secondly, the GTEx database has a small sample size, and this may account for differences between PRS_eQTLs_ deriving using GTEx and MetaBrain. Third, we limited our analyses to the expression quantitative trait loci (eQTLs), excluding other types of quantitative trait loci like (e.g. methylation quantitative trait loci (mQTLs) or protein quantitative trait loci (pQTLs)). Fourth, PRSs were built using effect sizes derived from GWAS on schizophrenia and not from a GWAS on early psychosis. When a robust GWAS on early psychosis becomes available, it will be important to update these analyses.

Notably, the main advantages in using early psychosis data are: (1) to avoid chronicity and long-term treatment that can be confounding factors for causal mechanisms, and (2) take advantage of the dynamic/plasticity of the early phases in order to modulate patient trajectories towards early detections and intervention or treatment [53, 54].

One current imperative of GWAS studies is to ‘translate’ the reported statistical genomic associations and to derive biological mechanisms; that is, to identify causal genes or ‘causal’ biological pathways [55] that underlie reported statistical genomic associations [56]. We reported here a reversed strategy, starting from known biological pathways which belong to a critical hub of schizophrenia pathophysiology, centered on redox dysregulation/ oxidative stress [12]. These biological pathways have been observed in numerous preclinical models based on genetic and environmental schizophrenia risk factors [49] and validated in patients [19, 57–64].

Our results highlight the critical role clinically-associated functional variants and the focus on specific pathways associated with the disease in the predictive accuracy with polygenic risk scores.

This could also represent a potential strategy towards defining cohorts based on individuals at high/low thresholds of pathway-specific PRS. As a pathway-specific score involves fewer variants, it could be more stable [65] and highlights interesting subsets of individuals for molecular/functional research, where the generic genome-wide “disease risk” score would be noisier. Taken altogether, the results from our analyses emphasize the need to build and study functionally informed risk scores which, after validation in larger cohorts, could improve the precision of patient stratification and personalized therapy.

## Supplementary information

Supplementary Figure 1

Supplementary Figure 2

Supplementary Table 1

Supplementary Table 2

Supplementary Table 3

Supplementary Table 4

Supplemetary Tables 5-9
