# Supplementary material for: Gene set enrichment analysis of pathophysiological pathways highlights oxidative stress in psychosis
Source: Mol Psychiatry. 2022 Sep 21;27(12):5135–43. doi: 10.1038/s41380-022-01779-1 (PMC9763118; doi:10.1038/s41380-022-01779-1)
Supplement: Supplementary file 2 — Supplementary Figure 2 [file 41380_2022_1779_MOESM2_ESM.docx]

**Supplementary Figure 2**


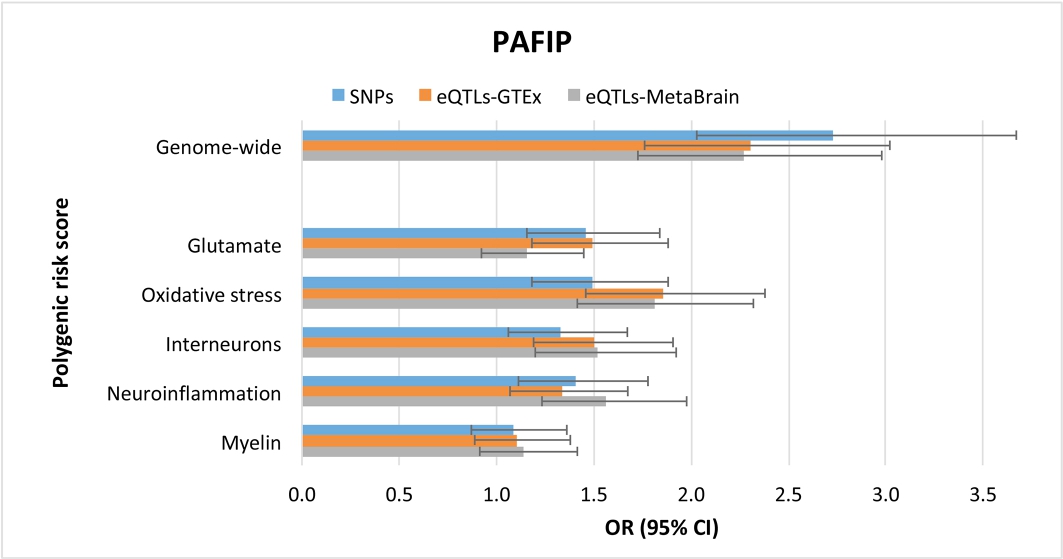


Results for the PAFIP study and polygenic risk scores (GW-PRSs and pathway-PRSs) analyses at pt ≤ 0.05. Early psychosis status (dependent variable) was regressed on the polygenic risk scores using logistic regressions and including the first five ancestry-informative genetic principal components as covariates. Horizontal bars show the Odds Ratio estimates (OR), and error bars indicate 95% confidence intervals (95% CI).
