# Supplementary material for: Gene set enrichment analysis of pathophysiological pathways highlights oxidative stress in psychosis
Source: Mol Psychiatry. 2022 Sep 21;27(12):5135–43. doi: 10.1038/s41380-022-01779-1 (PMC9763118; doi:10.1038/s41380-022-01779-1)
Supplement: Supplementary file 3 — Supplementary Table 1 [file 41380_2022_1779_MOESM3_ESM.docx]

**Supplementary Table 1. Demographic of participants in TIPP and PAFIP**

|  |  | **n** | **age (years)**  **[range]** | **Women %** |
| --- | --- | --- | --- | --- |
| **TIPP** | Early psychosis patients | 208 | 24.9  [18 – 35] | 32.2% |
|  | Controls | 132 | 29.8  [18 – 58] | 35.6% |
| **PAFIP** | Early psychosis patients | 224 | 30.5  [16 – 60] | 44.2% |
|  | Controls | 128 | 29.6  [15 – 50] | 37.5% |
