# Supplementary material for: Gene set enrichment analysis of pathophysiological pathways highlights oxidative stress in psychosis
Source: Mol Psychiatry. 2022 Sep 21;27(12):5135–43. doi: 10.1038/s41380-022-01779-1 (PMC9763118; doi:10.1038/s41380-022-01779-1)
Supplement: Supplementary file 4 — Supplementary Table 2 [file 41380_2022_1779_MOESM4_ESM.docx]

**Supplementary Table 2. Number of overlapping genes between pathways**

| Pathway | Glutamate | Oxidative stress | Interneurons | Neuroinflammation | Myelin |
| --- | --- | --- | --- | --- | --- |
| Glutamate N, (%) |  | 88 (14.3) | 138 (22.0) | 66 (10.5) | 30 (4.8) |
| Oxidative stress N, (%) | 88 (6.49) |  | 81 (6.0) | 179 (13.2) | 41 (3.0) |
| Interneurons N, (%) | 138 (10.3) | 81 (6.01) |  | 129 (9.6) | 31 (2.3) |
| Neuroinflammation N, (%) | 66 (4.0) | 179 (10.8) | 129 (7.8) |  | 24 (1.5) |
| Myelin N, (%) | 30 (15.4) | 41 (21.0) | 31 (15.9) | 24 (12.3) |  |

Total number of genes is 627 for Glutamate, 1,355 for Oxidative stress, 1,347 for Interneurons, 1,657 for Neuroinflammation and 195 for Myelin.

Abbreviations: N = number of overlapping genes; % = number of overlapping genes / total number of genes in the pathway.
