# Supplementary material for: Gene set enrichment analysis of pathophysiological pathways highlights oxidative stress in psychosis
Source: Mol Psychiatry. 2022 Sep 21;27(12):5135–43. doi: 10.1038/s41380-022-01779-1 (PMC9763118; doi:10.1038/s41380-022-01779-1)
Supplement: Supplementary file 6 — Supplementary Table 4 [file 41380_2022_1779_MOESM6_ESM.docx]

**Supplementary Table 4. Results for the PAFIP study and polygenic risk scores (GW-PRSs and pathway-PRSs) analyses at pt ≤ 0.05**

|  |  | OR | CI (95%) | Pval | Competitive Pval | N SNPs | Nagelkerke r^2^ | Liability-scale r^2^ | AUROC |
| --- | --- | --- | --- | --- | --- | --- | --- | --- | --- |
| eQTLs-GTEx | Genome-wide | 2.30 | [1.76 - 3.02] | 1.46E-09 * | - | 31,796 | 0.155 | 0.082 | 0.70 |
|  |  |  |  |  |  |  |  |  |  |
|  | Glutamate | 1.49 | [1.18 - 1.88] | 7.75E-04 * | 0.121 | 2,563 | 0.043 | 0.026 | 0.59 |
|  | Oxidative stress | 1.86 | [1.45 - 2.38] | 7.76E-07 * | 0.014 ** | 5,206 | 0.098 | 0.038 | 0.66 |
|  | Interneurons | 1.50 | [1.19 - 1.91] | 7.52E-04 * | 0.316 | 4,486 | 0.044 | 0.029 | 0.60 |
|  | Neuroinflammation | 1.34 | [1.07 - 1.68] | 0.011 | 0.837 | 6,001 | 0.024 | 0.016 | 0.58 |
|  | Myelin | 1.10 | [0.88 - 1.38] | 0.384 | 0.695 | 860 | 0.003 | 0.002 | 0.52 |
|  |  |  |  |  |  |  |  |  |  |
| eQTLs-MetaBrain | Genome-wide | 2.27 | [1.72 - 2.98] | 4.36E-09 * | - | 32,592 | 0.147 | 0.085 | 0.69 |
|  |  |  |  |  |  |  |  |  |  |
|  | Glutamate | 1.16 | [0.93 - 1.45] | 0.198 | 0.938 | 3,593 | 0.006 | 0.005 | 0.47 |
|  | Oxidative stress | 1.81 | [1.41 - 2.32] | 2.63E-06 * | 0.077 | 7,197 | 0.089 | 0.057 | 0.64 |
|  | Interneurons | 1.52 | [1.19 - 1.92] | 6.12E-04 * | 0.574 | 7,012 | 0.046 | 0.029 | 0.60 |
|  | Neuroinflammation | 1.56 | [1.23 - 1.98] | 2.44E-04 * | 0.583 | 8,289 | 0.053 | 0.030 | 0.62 |
|  | Myelin | 1.14 | [0.91 - 1.42] | 0.258 | 0.686 | 1,193 | 0.005 | 0.002 | 0.53 |
|  |  |  |  |  |  |  |  |  |  |
| SNPs | Genome-wide | 2.73 | [2.03 - 3.67] | 3.04E-11 * | - | 47,554 | 0.198 | 0.108 | 0.73 |
|  |  |  |  |  |  |  |  |  |  |
|  | Glutamate | 1.46 | [1.15-1.84] | 1.69E-03 * | 0.027 ** | 3,303 | 0.038 | 0.021 | 0.57 |
|  | Oxidative stress | 1.49 | [1.18-1.88] | 8.71E-04 * | 0.333 | 4,524 | 0.043 | 0.023 | 0.62 |
|  | Interneurons | 1.33 | [1.06-1.67] | 0.013 | 0.761 | 5,073 | 0.023 | 0.015 | 0.58 |
|  | Neuroinflammation | 1.41 | [1.11-1.78] | 4.33E-03 | 0.655 | 5,331 | 0.031 | 0.020 | 0.58 |
|  | Myelin | 1.09 | [0.87-1.36] | 0.457 | 0.770 | 975 | 0.002 | 0.001 | 0.52 |

Early psychosis status (dependent variable) was regressed on the PRSs using logistic regressions and including the first five ancestry-informative genetic principal components as covariates. Odds ratios (OR), 95% confidence intervals CI (95%) and p-value (Pval) show the predictive power of each PRS. Associations surviving correction for multiple testing alpha level of 0.0027 (0.05/18) are denoted with an asterisk (*). Number of SNPs included in the pathway analysis shows how many SNPs are included in each PRS. The column Competitive Pval shows the enrichment after 10,000 permutations. Significant enrichments are denoted with two asterisks (**). Nagelkerke r^2^ shows the variance explained by the PRS. Liability-scale r^2^ shows the variance explained by the PRS using a population prevalence of 0.7%. AUROC shows the area under the receiver operating characteristic curve.
